# Polarity and timing-dependent effects of transcranial direct current stimulation in explicit motor learning

**DOI:** 10.1016/j.neuropsychologia.2011.02.009

**Published:** 2011-04

**Authors:** C.J. Stagg, G. Jayaram, D. Pastor, Z.T. Kincses, P.M. Matthews, H. Johansen-Berg

**Affiliations:** a Centre for Functional Magnetic Resonance Imaging of the Brain (FMRIB), Nuffield Department of Clinical Neurosciences, University of Oxford, Oxford, UK; b Department of Biomedical Engineering, Johns Hopkins University School of Medicine, 720 Rutland Avenue, Baltimore, MD 21205, USA; c INSERM, U864, Espace et Action, 16 avenue Lépine, Bron F-69676, France; d Department of Clinical Neurosciences, Imperial College London, and GSK Clinical Imaging Centre, Hammersmith Hospital, London, UK

**Keywords:** Motor cortex, Human, Reaction times

## Abstract

Transcranial direct current stimulation (tDCS) is attracting increasing interest as a therapeutic tool for neurorehabilitation, particularly after stroke, because of its potential to modulate local excitability and therefore promote functional plasticity. Previous studies suggest that timing is important in determining the behavioural effects of brain stimulation. Regulatory metaplastic mechanisms exist to modulate the effects of a stimulation intervention in a manner dependent on prior cortical excitability, thereby preventing destabilization of existing cortical networks. The importance of such timing dependence has not yet been fully explored for tDCS. Here, we describe the results of a series of behavioural experiments in healthy controls to determine the importance of the relative timing of tDCS for motor performance. Application of tDCS *during* an explicit sequence-learning task led to modulation of behaviour in a polarity specific manner: relative to sham stimulation, anodal tDCS was associated with faster learning and cathodal tDCS with slower learning. Application of tDCS *prior to* performance of the sequence-learning task led to slower learning after both anodal and cathodal tDCS. By contrast, regardless of the polarity of stimulation, tDCS had no significant effect on performance of a simple reaction time task. These results are consistent with the idea that anodal tDCS interacts with subsequent motor learning in a metaplastic manner and suggest that anodal stimulation modulates cortical excitability in a manner similar to motor learning.

## Research highlights

► Transcranial direct current stimulation (tDCS) modulates explicit sequence learning. ► Anodal tDCS applied during the task speeds motor learning. ► Anodal tDCS applied before the task slows motor learning. ► Cathodal tDCS slows the rate of learning in both cases.

## 1 Introduction

Transcranial direct current stimulation (tDCS) is a non-invasive stimulation technique that allows the modulation of cortical excitability in humans in a polarity-specific manner. tDCS is attracting increasing interest as a neurorehabilitation tool for patients with chronic disability after stroke, in whom stimulation during performance of a motor task can lead to an improvement in motor function (Hummel & Cohen, 2005; Hummel et al., 2005, 2006).

In a healthy population anodal stimulation to the primary motor cortex (M1) leads to an increase in cortical excitability as evidenced by an increase in hand motor evoked potential (MEP) size, while cathodal stimulation leads to inhibition as assessed by a decrease in MEP amplitude. These neurophysiological effects outlast the stimulation period by up to 90 min (Nitsche & Paulus, 2000, 2001).

Behavioural effects of tDCS in healthy controls do not directly mirror these robust electrophysiological effects. Anodal tDCS applied to M1 during task execution improves performance in tests of motor speed and dexterity (Nitsche et al., 2003) and of motor learning and adaptation (Boggio et al., 2006; Galea & Celnik, 2009; Hunter, Sacco, Nitsche, & Turner, 2009; Nitsche et al., 2003; Reis et al., 2009). Cathodal tDCS, by contrast, has no effect on learning (Galea & Celnik, 2009; Nitsche et al., 2003; Reis et al., 2009) or on simple reaction times (Nitsche et al., 2003).

In addition, the behavioural effects of anodal stimulation depend on the relative timing of the stimulation and task. Concurrent anodal tDCS and performance of an implicit learning task lead to an improvement in the rate of learning of that task (Nitsche et al., 2003). However, when the task is performed after a period of stimulation, the rate of learning is reported to be unchanged (Kuo et al., 2008).

Understanding the interaction between tDCS and motor learning has important implications for developing rehabilitation approaches if the effects of tDCS may depend on the timing with which it is applied relative to physical training interventions. However, to the best of our knowledge, no study to date has compared responses with a procedural learning paradigm performed both during and after tDCS. In this study we investigate the timing-dependent effects of both anodal and cathodal tDCS on learning a specific sequence of finger movements.

## 2 Methods

Three cohorts of healthy volunteers were recruited with local ethics committee approval and all experiments were conducted in accordance with the Declaration of Helsinki. All subjects gave written, informed consent prior to their inclusion in the study. Seven volunteers (2 male; mean age 26 years [range 21–31]) participated in experiment 1. Seven volunteers (3 male; mean age 26 years [range 22–31]) participated in experiment 2. Eight volunteers (4 male; mean age 29 years [range 24–33]) participated in experiment 3. Two subjects participated in all experiments, one subject participated in both experiments 2 and 3. No subjects had any previous neurological or psychiatric history nor any contraindications to tDCS and all were right-handed as assessed by the Edinburgh Handedness Inventory (Oldfield, 1971). For all experiments, each subject had three testing sessions, during which they received anodal, cathodal or sham tDCS, in an order counterbalanced across the group. All stimulation sessions were separated by at least 48 h and all true stimulation sessions by at least 1 week.

Subjects were seated at a comfortable distance in front of a computer screen and performed a visually cued task consisting of sequential finger presses with their right hand. Four markers were displayed in the centre of the screen during the inter-trial interval. Each cue event consisted in one of the markers changing to an “x”. Subjects were instructed to press the button on a four button keypad that spatially corresponded to the position of the visual cue on the screen as quickly and accurately as possible with their right hands.

### 2.1 Experiment 1—reaction time task

Visual cues were presented in an unpredictable manner in the centre of computer screen in Arial font (bold, 36 point, grey [186:186:186]) on a background of darker grey (188:188:188). Cue duration was 9 ms, followed by the inter-trial display. The inter-trial interval was jittered pseudorandomly between 800 ms and 1800 ms. Each block started with the warning message “Get Ready” displayed in the centre of the screen for 1000 ms, followed by 30 cues in a pseudorandom order constrained to a ratio of 3:3:2:2. There was a 12 s inter-block interval.

Subjects performed three task blocks before stimulation. tDCS was then applied for 10 min. Immediately on cessation of the stimulation the 15 post-stimulation task blocks were commenced.

### 2.2 Experiments 2 and 3—explicit learning task

The task was identical for experiments 2 and 3. Visual cues were presented in a predictable manner in the centre of computer screen in Arial font (bold, 72 point, black on a background of white). Cue duration was 150 ms, followed by the inter-trial display. The inter-trial interval was jittered pseudorandomly between 1000 ms and 2000 ms. Each block started with the warning message “Get Ready” displayed in the centre of the screen for 1000 ms, followed by 3 repetitions of a 10-cue task constrained to a ratio of 3:3:2:2. There was a 12 s inter-block interval, giving a total duration of 15 min. Subjects were explicitly informed of the inherent sequence within the visual cues and were asked to memorize it and to respond as quickly and accurately as possible.

Three sequences of equal difficulty with the same ratio of digit presses (3:3:2:2) were presented in a counter-balanced order. The sequences were (1 = index finger, 2 = middle finger, 3 = ring finger and 4 = little finger) [3 1 2 4 2 1 3 1 2 4 2], [1 3 2 1 4 3 2 3 1 2] and [2 1 4 2 1 3 2 3 1 4]. In order to ensure that subjects had learnt the sequence they were asked to reproduce the sequence by repeating the order of button presses (in terms of 1–4) at the end of the experimental session. Data from subjects who were unable to accurately recall the sequence were excluded from further analysis.

### 2.3 tDCS

A DC-stimulator (Eldith GmbH; Germany) delivered a 1 mA current to the brain via 2 electrodes measuring 5 cm × 7 cm, one positioned 5 cm lateral and 2 cm anterior to Cz over the left hemisphere (the M1 electrode), and the reference positioned over the contralateral supraorbital ridge. This electrode configuration elicits the commonly reported neurophysiological effects whereby anodal tDCS increases and cathodal tDCS decreases MEP amplitude (Stagg, Best, et al., 2009). Water-soaked sponges were used as a conducting medium between the scalp and the electrodes. For true stimulation the current was ramped up over 10 s, held constant at 1 mA for 10 min and then ramped down over 10 s. For sham stimulation the current was ramped up over 10 s and then immediately switched off. Subjects are not able to distinguish between true and sham stimulation using this paradigm (Gandiga, Hummel, & Cohen, 2006), although we did not directly test this here.

In experiment 2 the task commenced 10 s after the tDCS current was turned on, and continued for 5 min after the end of the stimulation period (end of block 10). In experiments 1 and 3 the task commenced immediately on cessation of the tDCS. In experiments 1 and 3 subjects were seated at rest during stimulation, verbal interaction was kept to a minimum and they were instructed not to move their right hands.

### 2.4 Data analysis

Data from each subject were analysed on a block-by-block basis. For each block, any trials for which there were no response or for which the response was incorrect were deleted. In addition, any reaction times that deviated by more than ±2SD from the mean were excluded from the analysis. The mean and the standard deviation of the remaining reaction times for each block were calculated. The three pre-stimulation blocks were averaged to give a mean baseline reaction time.

For experiment 1 the mean reaction time (RT) was calculated for each block and was transformed into a change ratio by dividing it by the mean baseline RT (i.e. ΔRT = mean RT_Block_/baseline RT). For experiments 2 and 3 the mean RT for each block was transformed into a change ratio by dividing it by the mean RT from the first sequence (i.e. ΔRT = mean RT_Block_/first sequence RT).

## 3 Results

### 3.1 Experiment 1—reaction time task

This experiment was performed to test for behavioural effects of tDCS on reaction times with a simple cued reaction time task. The mean reaction time (RT) was calculated for each block and was transformed into a change ratio for that block (i.e. ΔRT = mean RT_Block_/baseline RT). A repeated measures ANOVA was conducted on the change in reaction time data with one factor of stimulation conditions (anodal, cathodal and sham) and one factor of time (15 blocks). There was a significant increase of reaction times over time (ANOVA *F*(14,70) = 2.87, *p* = 0.001), but no main effect of stimulation condition (ANOVA *F*(2,12) = 0.24, *p* = 0.78) or any interaction between time and stimulation (ANOVA *F*(28,168) = 0.97, *p* = 0.5 (Fig. 1)). Raw reaction time data is shown in Supplementary Fig. 1.

### 3.2 Experiment 2—explicit sequence learning task during tDCS

This experiment was conducted to investigate the behavioural effects of concurrent tDCS on performance during an explicit sequence learning task. In order to further exclude the possibility that stimulation effects on performance were due to an effect of stimulation on reaction times rather than on learning, the mean reaction time was calculated for each block and was transformed into a change ratio from the reaction time for the first sequence (i.e. ΔRT = mean RT_Block_/first sequence RT). A repeated measures ANOVA was conducted on the change in reaction time data with one factor of stimulation condition (anodal, cathodal and sham) and one factor of time (15 blocks).

There was a significant shortening of reaction times across time in all stimulation conditions, consistent with learning the sequence presented (ANOVA main effect of block (*F*(14,84) = 7.35, *p* < 0.001) (Fig. 2). There was no main effect of stimulation condition (ANOVA (*F*(2,12) = 0.89, *p* > 0.4), but there was a significant interaction between time and stimulation condition, suggesting that learning rates varied between stimulation conditions (ANOVA (*F*(28,168) = 2.87, *p* = 0.001).

Subsequent planned ANOVAs were performed to separately contrast each stimulation condition to sham. Contrasting anodal tDCS and sham revealed no main effect of stimulation (*F*(1,6) = 0.162, *p* > 0.46), but there was a significant interaction between stimulation condition and time (*F*(14,84) = 2.99, *p* = 0.001). Comparing cathodal tDCS to sham revealed a significant main effect of stimulation condition (*F*(1,6) = 4.78, *p* = 0.04); reaction times *increased* with cathodal stimulation. There also was a significant interaction between stimulation condition and time (*F*(14,84) = 1.76, *p* = 0.03).

In addition, we directly compared anodal and cathodal stimulation. There was no main effect of stimulation (*F*(1,6) = 0.48, *p* > 0.5) but there was a significant interaction between stimulation condition and time (*F*(14,84) = 3.19, *p* = 0.001).

Comparable results were found using non-normalized data (see Supplementary results). No effects on accuracy were observed (see Supplementary results).

### 3.3 Experiment 3—explicit sequence learning task after tDCS

This experiment was conducted to investigate the behavioural after-effects of tDCS on an explicit learning task which commenced after the stimulation has ceased. One subject was not able to reproduce the sequence verbally at the end of the experiment on any occasion and was therefore excluded from further analysis.

The mean reaction time was calculated for each block and was transformed into a change ratio from the reaction times to the first sequence (i.e. ΔRT = mean RT_Block_/first sequence RT). A repeated measures ANOVA was conducted on the change in reaction time data with one factor of stimulation condition (anodal, cathodal and sham) and one factor of time (15 blocks).

There was a significant shortening of reaction times over time across all conditions (ANOVA *F*(14,84) = 10.33, *p* < 0.01) (Fig. 3). There was also a significant effect of tDCS condition on reaction times (ANOVA *F*(2,12) = 4.24, *p* < 0.05), but no significant interaction between block and stimulation condition (ANOVA *F*(28,198) = 1.18, *p* = 0.25).

Subsequent planned ANOVAs demonstrated a significant *increase* in reaction times with anodal stimulation compared to sham (*F*(1,6) = 3.87, *p* = 0.04), but no significant interaction between stimulation condition and time (*F*(14,84) = 1.52, *p* = 0.11). There was also a significant increase in reaction times with cathodal stimulation compared to sham (*F*(1,6) = 7.54, *p* = 0.03), though again there was no interaction between stimulation condition and time (*F*(14,84) = 1.42, *p* = 0.15).

In addition, we compared anodal and cathodal stimulation. There was no difference in response between the two conditions (*F*(1,6) = 0.279, *p* > 0.6) nor any interaction between stimulation condition and time (*F*(14,84) = 0.732, *p* > 0.7).

Comparable results were found using non-normalized data (see Supplementary results). No effects were found on accuracy (see Supplementary results).

### 3.4 Comparison between the effects of stimulation applied before (experiment 2) or during (experiment 3) task performance

In order to directly investigate the timing-dependent differences in the effects of tDCS, we compared the data on change in reaction times from experiments 2 and 3 for each stimulation condition separately. There was no difference between reaction time change ratio in the two learning experiments with sham stimulation (*F*(1,13) = 0.10, *p* > 0.7). There was a significant difference between the rates of change in reaction times when the tDCS was applied before and during the motor task for anodal stimulation, but no difference for cathodal stimulation (anodal tDCS [*F*(1,13) = 4.8, *p* = 0.03], cathodal tDCS [*F*(1,13) = 0.18, *p* > 0.6]). Specifically, anodal stimulation during task performance (experiment 2) was associated with greater reaction time change ratios (i.e. faster learning) than anodal stimulation applied before task performance (experiment 3).

## 4 Discussion

This study was performed to investigate the timing-dependent interactions between tDCS and learning of an explicit-learning paradigm. In order to characterize these interactions we studied the effects of tDCS on reaction times in a sequence learning task both when tDCS was applied during the task and when it was applied prior to performance of the task. tDCS modulated learning rates in all conditions. Stimulation applied during motor practice modulated learning rates in a polarity-specific manner; anodal tDCS *increased* the rate of motor sequence learning while cathodal stimulation *decreased* the rate of learning. Either anodal or cathodal tDCS applied prior to the motor task led to a slowing of learning when compared to sham stimulation.

Motor learning is dependent on Hebbian synaptic plasticity mechanisms, such as long-term potentiation (LTP)-like changes, within the interneurons of the primary motor cortex (Muellbacher et al., 2002; Stefan et al., 2005; Ziemann, Iliac, Pauli, Meintzschel, & Ruge, 2004). LTP-like plasticity operates by positive feedback and therefore carries the potential to destabilize established cortical networks, leading to unregulated cortical activity and preventing further dynamic modifications (Abbott & Nelson, 2000). In order to prevent this destabilization, regulatory metaplasticity mechanisms have been proposed to operate (Bienenstock, Cooper, & Munro, 1982; Sejnowski, 1977) to maintain neural activity within a useful range. Metaplasticity has been demonstrated in humans, when the effects of a train of transcranial magnetic stimulation (TMS) pulses normally insufficient to induce excitability changes become inhibitory if applied after anodal tDCS, and become excitatory if applied after cathodal tDCS (Lang et al., 2004; Siebner et al., 2004).

A parsimonious explanation for the timing-dependent interaction between anodal tDCS and motor learning demonstrated here, therefore, is that of metaplastic mechanisms. This explanation would be partially in line with a previous study showing that prior application of anodal tDCS slowed subsequent motor learning, although in that case effects were only seen with the application of a partial NMDA-receptor agonist (Kuo et al., 2008). The discrepant findings of the effects of anodal tDCS alone may reflect differences in the sensitivity and demands of the different tasks used in the two studies.

In contrast to our findings with anodal stimulation, the relative timing of stimulation and task had no bearing in the effects of cathodal stimulation. Metaplastic mechanisms are usually network specific, i.e. a prior modulatory stimulus (for example tDCS) will only modify the response to a subsequent one (for example, motor learning) if these two stimuli involve the same groups of circuits and synapses (Abraham, Mason-Parker, Bear, Webb, & Tate, 2001). The lack of an interaction between cathodal tDCS and motor learning suggests, therefore, that cathodal tDCS and motor learning affect motor cortical plasticity via distinct mechanisms.

The hypothesis that anodal and cathodal tDCS modulate distinct neuronal populations is consistent with our recent study using magnetic resonance spectroscopy that demonstrated that anodal tDCS decreased GABA within the stimulated M1 (Stagg, Best, et al., 2009), a change similar to that observed during motor learning (Floyer-Lea, Wylezinska, Kincses, & Matthews, 2006). By contrast, cathodal tDCS affected glutamate levels, which have not been reported to change with motor learning in the same way (Stagg, Best, et al., 2009), and the addition of the GABA agonist lorazepam has no effect on the after-effects of cathodal stimulation, although it does modulate the after-effects of anodal stimulation (Nitsche et al., 2004). Previous functional MRI studies also suggest that anodal and cathodal stimulation modulate distinct systems-level networks within the active motor system (Stagg, O'Shea, et al., 2009).

The speeding of explicit learning with on-line anodal stimulation is in line with previous studies that have demonstrated speeding of learning of an implicit sequence-learning paradigm with anodal tDCS (Nitsche et al., 2003; Reis et al., 2009). However, while we found slowing of learning with (both on-line and off-line) cathodal tDCS, consistent with its inhibitory neurophysiological effects, such a slowing effect on learning was not found in previous studies (Galea & Celnik, 2009; Nitsche et al., 2003; Reis et al., 2009). The reasons for this discrepancy are unclear. It may be that the implicit tasks used in the previous studies are less demanding of M1, so that the decrease in cortical excitability induced by cathodal tDCS has no behavioural consequences. If the explicit learning task used here is more demanding on the cortex, then the decrease in excitability induced by cathodal tDCS may lead directly to a decrease in functional outcomes as there is insufficient redundancy in the system. However, this conclusion has yet to be tested directly.

It is possible that the slowing in motor learning observed after both anodal and cathodal tDCS merely reflects addition of “noise” into the system, interfering with the long distance coherence in network activity important for motor learning, in a manner similar to that induced by low-frequency rTMS (Strens et al., 2002). However, this explanation seems less likely given that there is no change in accuracy after true stimulation compared with sham; that concurrent stimulation leads to behavioural improvements (Boggio et al., 2006; Galea & Celnik, 2009; Nitsche et al., 2003; Reis et al., 2009) and that specific behavioural effects are unmasked by application of a NMDA partial agonist following anodal stimulation (Kuo et al., 2008).

Consistent with a previous report (Kuo et al., 2008), we did not find any effects of off-line tDCS on simple reaction times in the current study, although another study has reported on-line simple reaction time effects (Nitsche et al., 2003). To rule out the possibility that our learning effects depend on changes in reaction times due to tDCS, the results from both learning experiments presented are normalized to the reaction times during the first sequence performed after tDCS. Therefore, our measures of change in motor learning are corrected for any overall change in reaction time due to tDCS.

tDCS may effect learning in two ways: it may decrease the total *amount* of learning achieved, such that different minimum RTs are reached, or it may decrease the *rate* at which learning is achieved, such that the same ultimate RTs are achieved, but over a different timescale. In this study tDCS modulates both the total amount of learning and the rate at which learning is achieved. Specifically, anodal stimulation applied during motor learning increases the *rate* of learning, but does not affect the ultimate amount of that learning (seen as a stimulation × time interaction, rather than a main effect of stimulation in the ANOVA analyses). Conversely, cathodal stimulation leads to a decrease in both the rate and the amount of learning. These findings suggest that a single-session of tDCS, as applied here, cannot modulate the total amount of learning achieved.

We have only investigated the effects of tDCS on learning over short periods. Other studies have suggest that tDCS also has effects on the consolidation of motor learning (Reis et al., 2009), and it would be interesting to retest subjects at a later time point to investigate whether later effects are also dependent on the relative timing of tDCS and the learning task. In particular, both off-line stimulation conditions and on-line cathodal stimulation show a main effect of stimulation compared with sham across the entirety of the motor task, whereas there is an interaction between time and on-line anodal stimulation suggesting that the effects of this stimulation paradigm have decreased in relative terms by the end of the stimulation period. This distinction may be important for understanding later after-effects.

This study was performed to examine the relationship between the timing of tDCS and motor learning. The finding that prior application of anodal tDCS slows subsequent motor learning is important in the context of neurorehabilitation. Explicit sequence learning in healthy subjects involves many of the same underlying processes that are also important for motor rehabilitation after chronic stroke (Krakauer, 2006). Given the increasing interest in the potential clinical utility of tDCS for motor improvements in rehabilitation (Hummel & Cohen, 2006), the current study suggests that anodal tDCS should be applied *during* a physiotherapy intervention for its effects to be maximally beneficial.

## Figures and Tables

**Figure:**
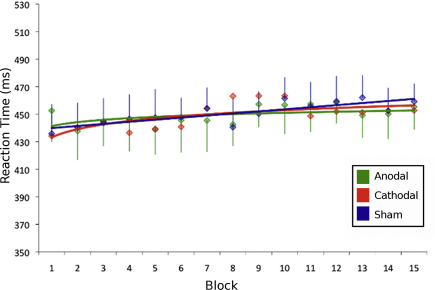


**Figure:**
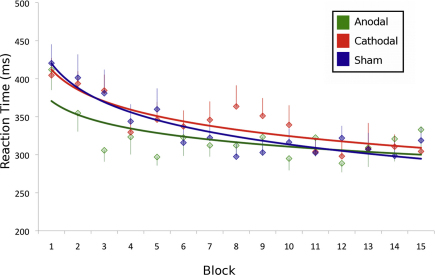


**Figure:**
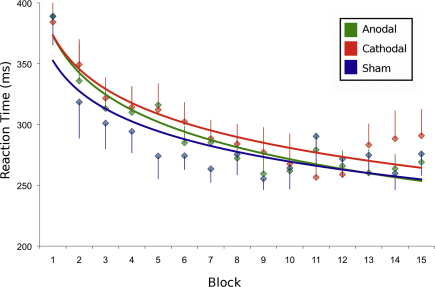


**Fig. 1:**
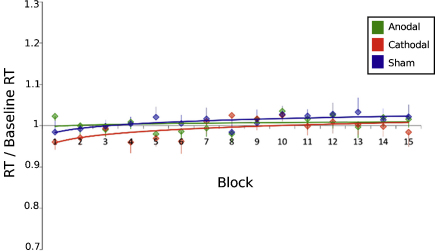
Reaction times in response to the simple response task, normalized with respect to the mean response time during the baseline blocks. A logarithmic trend-line is superimposed for clarity. No difference in reaction times between stimulation conditions can be seen (mean ± SE).

**Fig. 2:**
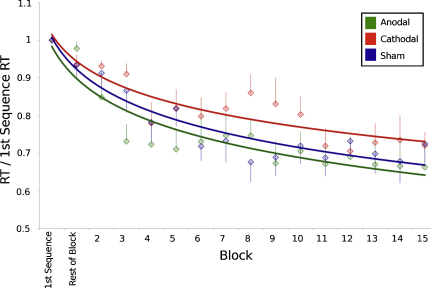
Mean reaction times in response to the learning task performed during tDCS, normalized to the reaction times for the first repetition of the sequence. A logarithmic trend line for each stimulation condition is superimposed for clarity. A significant speeding in the rate of learning is seen with anodal stimulation and a significant increase in reaction times is seen with cathodal tDCS (mean ± SE).

**Fig. 3:**
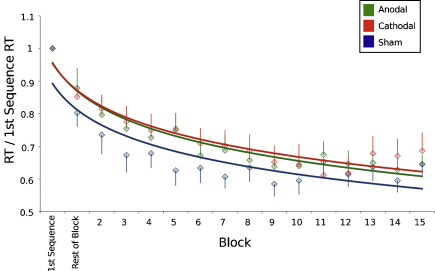
Mean reaction times in response to the learning task performed after tDCS, normalized to the reaction times for the first repetition of the sequence. A logarithmic trend-line for each stimulation condition is superimposed for clarity. A significant slowing is seen in the rate of learning following both anodal and cathodal stimulation compared with sham (mean ± SE).
