# Supplementary material for: Polarity and timing-dependent effects of transcranial direct current stimulation in explicit motor learning
Source: Neuropsychologia. 2011 Apr;49(5):800–4. doi: 10.1016/j.neuropsychologia.2011.02.009 (PMC3083512; doi:10.1016/j.neuropsychologia.2011.02.009)
Supplement: Supplementary file 1 [file mmc1.doc]

# Supplementary Information

## Supplementary Results

Results in the main manuscript are presented normalised either to the baseline reaction times in the case of experiment 1, or to the first sequence of a task in the case of experiments 2 & 3. In these supplementary results we provide these data and analyses as raw reaction times, for ease of interpretation. These results are broadly similar to those performed on the normalised data presented in the main body of the paper.

### Experiment 1

This experiment was performed to test for behavioural effects of tDCS on reaction times with a simple cued reaction time task. A repeated measures ANOVA was conducted on the reaction time data with one factor of stimulation conditions (anodal, cathodal and sham) and one factor of time (15 blocks). There was a significant increase of reaction times over time (ANOVA F(14,84) = 2.68, p = 0.003), but no main effect of stimulation condition (ANOVA Main effect of stimulation; F(2,12) = 0.16, p = 0.85) or any interaction between time and stimulation (ANOVA F(28,168) = 0.97, p = 0.5 (supplementary figure 1)).

### Experiment 2

This experiment was conducted to investigate the behavioural effects of concurrent tDCS on performance during an explicit sequence learning task. There was a significant shortening of reaction times across time in all stimulation conditions, consistent with learning the sequence presented (ANOVA main effect of block (F(14,84) = 8.82, p < 0.001) (figure 2). There was no main effect of stimulation condition (ANOVA (F(2,12) = 1.57, p > 0.2), but there was a significant interaction between time and stimulation condition, suggesting that learning rates varied between stimulation conditions (ANOVA (F(28,168) = 2.76, p = 0.001).

Subsequent planned ANOVAs were performed to separately contrast each stimulation condition to sham. Contrasting anodal tDCS and sham revealed no main effect of stimulation (F(1,6) = 0.61, p > 0.4), but there was a significant interaction between stimulation condition and time (F(14,84) = 2.96, p = 0.001). Comparing cathodal tDCS to sham revealed no significant main effect of stimulation condition (F(1,6) = 1.88, p = 0.219); reaction times *increased* with cathodal stimulation. There also was a significant interaction between stimulation condition and time (F(14,84) = 2.27, p = 0.01).

In addition, to investigate for any polarity-specific effects we directly compared anodal and cathodal stimulation. There was no main effect of stimulation (F(1,6) =2.50, p > 0.1) but there was a significant interaction between stimulation condition and time (F(14,84)=2.98, p =0.001).

Overall, there was no difference in mean accuracy between the baseline blocks and the learning blocks (ANOVA main effect of task (baseline vs learning) [F(1,6)=0.41, p > 0.8] and no interaction between stimulation condition and task [F(2,12)=0.47, p > 0.6]. In addition, we investigated accuracy across the learning task. There were no changes in the number of correct responses over the learning period (ANOVA main effect of block [F(14,84) = 1.18, p > 0.3]) no difference in accuracy rates between stimulation conditions [ANOVA F(2,12) = 1.1, p > 0.7], and no interaction between time and stimulation conditions [ANOVA F(28,168) = 0.77, p > 0.7].

### Experiment 3

There was a significant shortening of reaction times over time across all conditions (Repeated measures ANOVA main effect of block F(14,84) = 10.93, p < 0.01). There was also a significant effect of tDCS condition on reaction times (ANOVA main effect of tDCS F(2,12) = 4.38, p < 0.05), but no significant interaction between block and stimulation condition (ANOVA F(28,198) = 1.05, p = 0.4).

Subsequent planned ANOVAs demonstrated a significant *increase* in reaction times with anodal stimulation compared to sham (F(1,6) = 3.85, p = 0.04), but no significant interaction between stimulation condition and time (F(14,84) = 1.11, p = 0.35). There was also a significant increase in reaction times with cathodal stimulation compared to sham (F(1,6) = 6.23, p = 0.04), though again there was no interaction between stimulation condition and time (F(14,84) = 1.52, p = 0.12).

To determine whether these effects were polarity specific we compared anodal and cathodal stimulation. There was no difference in response between the two conditions (F(1,6) = 0.61, p > 0.5) nor any interaction between stimulation condition and time (F(14,84) = 0.732, p > 0.8).

### Comparison between the effects of stimulation applied before (experiment 2) or during (experiment 3) task performance

In order to directly investigate the timing-dependent differences in the effects of tDCS, we compared the data on change in reaction times from experiments 2 & 3 for each stimulation condition separately. There was no difference between reaction time change ratio in the two learning experiments with sham stimulation (F(1,13) = 4.43, p > 0.05). There was a significant difference between the rates of change in reaction times when the tDCS was applied before and during the motor task for anodal stimulation, but no difference for cathodal stimulation (Anodal tDCS [F(1,13) = 4.66, p = 0.03], cathodal tDCS [F(1,13) = 3.36, p = 0.09]). Specifically, anodal stimulation during task performance (experiment 2) was associated with greater reaction time change ratios (i.e., faster learning) than anodal stimulation applied before task performance (experiment 3).

## Figure Legends

### Supplementary Figure 1

Reaction times in response to the simple response task. No difference in reaction times between stimulation conditions can be seen (mean ± SE). A logarithmic trend-line is superimposed for clarity.

### Supplementary Figure 2

Mean reaction times in response to the learning task performed during tDCS. A logarithmic trend line for each stimulation condition is superimposed for clarity.

### Supplementary Figure 3

Mean reaction times in response to the learning task performed after tDCS. A logarithmic trend-line for each stimulation condition is superimposed for clarity.
